# Improving Dementia Clinical Research Participation: Strategies From a Portland, Oregon, Pilot Study

**DOI:** 10.1093/geroni/igaa057.187

**Published:** 2020-12-16

**Authors:** Nicole Bouranis, Sherril Gelmon, Elizabeth Needham Waddell, Dawn Richardson, Hyeyoung Woo, Allison Lindauer

**Affiliations:** 1 Oregon Health & Science University-Portland State University School of Public Health, Portland, Oregon, United States; 2 Portland State University, Portland, Oregon, United States; 3 Oregon Health & Sciences University, Portland, Oregon, United States

## Abstract

The NIA’s strategy to improve ADRD clinical research participation emphasizes local community collaboration. Literature that focuses on a person with dementia’s decision to participate in research does not speak to specific state or local factors nor the effects of local efforts. This study aimed to develop strategies to improve dementia research participation in the Portland, OR metropolitan area. A community advisory board comprised of clinicians, researchers, advocates, people with dementia, family caregivers, and older African Americans was established for this project. Thirty-three interviews were conducted with clinicians, researchers, advocates, people with ADRD, and family caregivers. The Robert Wood Johnson Foundation’s Culture of Health Action Framework was used to conceptualize motivation strategies and reflect elements that describe research participation among people with dementia. Strategies were identified to improve dementia clinical research participation: 1) Identify and promote local champions for ADRD clinical research participation; 2) Promote policies and processes that incentive cross-sector collaboration; 3) Recognize caregivers as full research participants; 4) Include people with ADRD and caregivers in the research design process; 5) Offer alternative options to reduce participation burden; 6) Evaluate and improve relationships between healthcare/research staff and patients/participants. These strategies can be used in conjunction with the Culture of Health Action Framework as a roadmap to form organization-community partnerships, facilitate motivation and empowerment, give decision-making power to people with ADRD and promote a local culture of research. Studies should be conducted in a larger context or as pilots in other communities to determine contextual relevance and generalizability for other areas.

